# Being Intelligent with Emotions to Benefit Creativity: Emotion across the Seven Cs of Creativity

**DOI:** 10.3390/jintelligence10040106

**Published:** 2022-11-15

**Authors:** Daniel Sundquist, Todd Lubart

**Affiliations:** Laboratoire de Psychologie et d’Ergonomie Appliquées (LaPEA), Université Paris Cité and Univ Gustave Eiffel, 92774 Boulogne-Billancourt, France

**Keywords:** creativity, emotional intelligence, emotional creativity

## Abstract

In this review of emotion, emotional intelligence (EI) and creativity, we look at the various ways that these topics can be explored together using the seven Cs of Creativity as a structuring framework. The seven Cs of creativity are: creators, creating, collaborations, contexts, creations, consumption and curricula, representing the different facets of creativity research. The question of emotion and creativity has a long historical lineage, which has led up to the study of intelligent and dynamic aspects of emotion and their impact on creativity. Previous and emerging work on EI, related emotional aspects and creativity offer promising ways to advance this field of research. However, we show that some aspects of creativity and EI are less explored than others. We offer several implications for the direction of future work.

## 1. Introduction

In early work on emotions in creativity, Freud (1958) posited that inner conflicts can be reworked subconsciously and re-emerge through creativity in a loosely associated but socially acceptable version. The act of creativity via this defense mechanism (called *sublimation*) would thereby be an adaptive way of regulating potentially overwhelming emotions, as compared to other forms of potentially psychopathological expression that forbidden wishes or self-incompatible emotions can take. 

Maslow (1958) described creativity as an act of self-actualization, “a kind of permission to be ourselves” (Maslow 1958, p. 51). In Maslow’s view, a hallmark of the creative person is the ability to produce a creative outcome “in great bursts of emotion and enthusiasm”, not critically evaluating the idea until a later stage (p. 57). He theorized that creativity would become inhibited from not being in touch with one’s fundamental emotions and drives (primary processes), which can happen, for example, in people suffering from compulsive–obsessive disorders. Rather than a *compromise* toward your inner emotions, as Freud described it, creativity is seen by Maslow as a way of being wholly true to who you are and what you feel. When something moves an individual toward “greater psychological health or fuller humanness”, then this global change should result in more creative behavior globally (Maslow 1972, p. 288).

Both of these examples illustrate the role of emotion in creativity being discussed in terms of the psychopathology vs. psychological well-being dimension. Early work on creativity also examined emotion-related aspects of the creative process, mainly in the context of eminent creativity. Famous creators have for a long time mentioned emotions as an integral part of the creative process, including them as a motivator, inhibitor, and as a sort of building material from which creations are made (e.g., Ghiselin 1952). In an introspective exploration of the sequence of steps of his own creative act, the French mathematician Poincaré described an *aesthetic sensitivity* working automatically as a screening mechanism (Poincaré [1908] 1985). This emotional mechanism would privilege ideas (or combinations of elements) that are perceived as beautiful to pass into consciousness, as these are also the ones that are most valuable mathematically. Across several case studies on literary creation, Binet included a number of emotional aspects, such as emotional characteristics of the authors’ personalities as well as creative processes (Binet and Passy 1895; Binet 1904). For example, contrasting two contemporary authors, Binet described one as working in a feverish, frenzied state, but only when struck by the feeling of inspiration. The second author remained calm and poised throughout the process, emotionally unburdened by stressors such as approaching deadlines. Hence, the latter was able to engage in his writing calmly for a designated time each day. 

More recently, the span of what constitutes creativity and who is capable of it have increased. The focus turned toward experimental studies on mood states and their effects on creativity (e.g., Baas et al. 2008). This shift was made possible due to methodological advances, such as the mood-induction paradigm as well as questionnaires and tests assessing various aspects of emotion and creativity. 

Current research extends the topic of emotion and creativity to include intelligent and dynamic aspects of emotion. One line concerns the impact of idiosyncratic emotional experience on creativity (Getz and Lubart 2000; Averill 1999). Another promising one is the impact of emotion-related abilities on creativity. The objective of this paper is to provide a conceptual review of the state of research, covering some selected, fruitful lines of research in each of the seven Cs of creativity (Lubart 2017). These will be illustrated with recent examples of empirical studies. The seven Cs of creativity are: creators, creating, collaborations, contexts, creations, consumption and curricula, representing the different facets of creativity research. This framework was initially constructed to cover the scientific literature on creativity after a survey of 50 years of articles published in the *Journal of Creative Behavior* (JCB). It expands upon the four Ps framework (person, process, press and product), which marked modern creativity research when published by Mel Rhodes in his 1961 article entitled “An analysis of creativity” (Rhodes 1961). The seven Cs for the study of creativity will be used as a structuring framework for illuminating intelligent and dynamic influences of emotion on creativity. 

## 2. Creators

The term *creators* concerns the person-centered characteristics in creative individuals (Lubart 2017). For the present paper, it relates to measurable individual differences on aspects of emotion, such as emotional intelligence (EI) and emotional creativity (EC). 

EI can be defined as the ability to accurately perceive emotions, to use emotions for assisting your thinking, to understand emotions and to regulate emotions in oneself and in others (Mayer and Salovey 1997). It can be studied in terms of self-reported individual differences—*trait EI*—or via performances on specific tests—*ability EI* (see Mayer et al. 2008). Despite some partially divergent findings in the past, a recent meta-analysis reported a moderately strong, positive correlation (r = 0.32) between EI and creativity (Xu et al. 2019). Moderator analyses show that the link was strongest in trait EI (r = 0.35) and creative personality (r = 0.33) and behavior (r = 0.38) and weaker but still statistically significant in divergent thinking tests (r = 0.07). Nonsignificant moderators were ability EI (r = 0.08), remote associate tests (r = 0.11) and ratings of creative products (r = 0.03), but the sample size was smaller in these three instances (ranging from 2 to 10 studies as opposed to 14 to 66 in the statistically significant moderators cited above). 

This line of research considers that people have agency when it comes to their own and others’ emotions, making it possible to influence and use emotions for creative ends (Ivcevic et al. 2021). Parke et al. (2015) suggest that workers high in EI efficiently generate and maintain positive affect, beneficial for creativity, when faced with high demands on information processing. Furthermore, when the job does not explicitly require creativity, high-EI individuals “regulate their responses to the boredom or lack of interest (...) by using effective emotion regulation strategies”, for example, by ”incorporating more creative behaviors (e.g., exploration and experimentation) in their tasks not formally prescribed” (p. 921). These individuals also know when to perform tasks that benefit from their positive mood state (e.g., requiring openness and divergent thinking), and they manage to stay motivated while doing so (Parke et al. 2015). The creative benefits of EI apply not only to positive but also negatively valenced mood states, for example, by allowing creators to “channel negative affect into finding alternative solutions” (Xu et al. 2019, p. 17). 

Individuals not only have agency in regard to processing, understanding and regulating emotions but can also be considered as creators of their own emotional experience and way of expressing it (Averill 1999). Averill (1999) states that there are measurable individual differences in the ability to learn from and understand emotions (*preparedness*) and in the extent to which your experienced emotions are unique (*novelty*) as well as appropriate and reflecting your true self (*effectiveness/authenticity*). EC as a construct designates the originality, appropriateness and authenticity of an individual’s way of experiencing and expressing emotions (Averill and Thomas-Knowles 1991) and is independent of EI (Ivcevic et al. 2007). Just like EI, EC can be assessed through self-report measures or by performance-based tests (Ivcevic et al. 2007). Both types of EC predict the type and amount of creative leisure activities that people engage in (Trnka et al. 2016; Ivcevic et al. 2007), but only self-reported EC is related to creative performance (Ivcevic et al. 2007; Averill 1999). Fuchs et al. (2007) also found a positive correlation between self-reported EC and self-reported creative capacities in participants. 

EC has been described as mainly facilitating *mini-c* activities with little impact on society (Ivcevic et al. 2017; Beghetto and Kaufman 2013). However, Fong (2006) provided evidence that experiencing emotions that do not usually “go together” can increase individuals’ potential to make unusual associations. Using emotion induction on participants who then completed the Remote Associate Test (RAT) (Mednick 1962), the emotionally ambivalent group outperformed the positive, negative and neutral valence groups. Being in an emotionally ambivalent state would signal that the current environment/situation is an unusual one and, thereby, that there are potentially new associations to make. The author argues that this could benefit creativity in an organizational setting (Fong 2006). As the tendency to experience a novel mix of emotions is one of the defining markers of emotionally creative people (Averill and Thomas-Knowles 1991), it is possible that EC could support creativity beyond the personal level of mini-c in a similar way. 

Ivcevic and Brackett (2015) have found that emotion regulation ability (a strand of EI) predicted peer-nominated ratings of creativity in a high school sample only when self-assessed openness to experience was high. This suggests that certain characteristics in the creator are required in order for EI to enhance creativity (Ivcevic and Brackett 2015). We consider EI and EC as belonging to the emotional factors that, alongside environmental, cognitive and conative factors, make up the profile of creative potential in an individual in a multivariate approach (Lubart et al. 2015). These factors will interact differently with each other according to the task at hand. Following this view, EI requires a cocktail of other individual factors in order to lead to a creative outcome, and this cocktail will differ between creative domains, tasks or stages in the creative process (e.g., Caroff and Lubart 2012).

## 3. Creating

*Creating* refers to the creative process: the sequence of steps, thoughts and actions that leads to a creative outcome (Lubart 2017). Emotions are tightly interwoven with the act of creation across many domains and throughout the creative process (Glaveanu et al. 2013). We will examine several ways that emotions can be used intelligently in the creative process. One way is employing emotional information stored in memory to make idiosyncratic associations. Second, emotional states can be actively monitored and manipulated in order to facilitate creativity. 

According to the emotional–experiential perspective on creative associations and metaphors, every individual possesses emotional information based on their own subjective life experiences (Getz and Lubart 2000). There is a rich network of emotional traces attached to images or concepts in memory. These representations, or emotional schemas, are referred to as *endocepts*. They are “acquired through people’s self-involving experiences and reflecting their covert subjective judgments and attitudes related to these experiences” (p. 290). The way that endocepts are idiosyncratically associated in memory could be compared to crystallized intelligence in that it consists of the individual’s emotional (as opposed to semantic) information about the world. A rich network of emotional information can be used in the generation as well as the interpretation of new ideas. The activation process is called “emotional resonance”, in which endocepts attached to a source concept will harmonically resonate with endocepts showing a similar emotional trace, although they may be attached to cognitively distant “target” concepts (Lubart and Getz 1997). Getz and Lubart (2000) traced participants’ production of creative metaphors for significant life events and showed that the metaphors were generated using idiosyncratic emotional traces. They also found that the same emotion-based process can be used when interpreting metaphors in new and creative ways that are both original and relevant to the individual. Emotion-based associations can thus be used for engaging in different stages of the creative process (generation and interpretation). It can be hypothesized that individuals with high levels of EC have a wider foundation of unique, idiosyncratic associations to draw from, given their tendency to experience unique blends of emotions in different situations (Averill and Thomas-Knowles 1991). 

In a series of experiments, Cohen and Andrade (2004) showed that people have intuitive theories on which mood state favors creative thinking (positive mood) as opposed to analytical thinking (negative mood). When told that they would perform a creativity task, the participants chose to put themselves in a happier mood by listening to a song with a happy-sounding title, and they did so explicitly in order to improve their creative performance. It also seems like these intuitive theories have multiple layers, involving emotion-related personality traits (Leung et al. 2014). When faced with an upcoming creativity task, individuals who scored high on trait neuroticism (i.e., who regularly have negative mood states such as worry or depression) showed a significantly higher tendency to put themselves in a negative mood by choosing to recall worrisome events (Leung et al. 2014). Participants in a congruent trait neuroticism and negative mood state also performed better on idea generation tasks. 

Interview studies of creators in the domains of art, design, science, scriptwriting and music have identified emotions that typically occur in different stages of the creative process (Glaveanu et al. 2013; Bourgeois-Bougrine et al. 2014). Emotion thus plays different roles according to the phase of the creative act as well as the domain in question (Glaveanu et al. 2013). Botella et al. (2011) traced the creative process as well as the emotional states of art students in two studies throughout a creative project spanning five days. At five points each day, the students filled out forms assessing their current emotional state (valence and arousal) as well as the stages they had engaged in since the previous evaluation. It was found that the creative process is not linear but dynamic, and that each phase is associated with an emotional profile including both negatively and positively valenced emotions of varying intensity. There were some preliminary trends concerning emotional differences in both studies between those who performed creatively above average (C+) and those below average (C+), suggesting that specific emotion states could be more or less beneficial in a given phase (Botella et al. 2011). If that is the case, then there could also be room for potential EI effects. In interviewing creative designers, Sas and Zhang (2010) found that these designers were highly familiar with their own emotional responses along the different steps of the creative process. The creative designers reported using this knowledge to boost creativity, for example, by putting themselves in a positive mood before brainstorming or inhibiting strong emotions during the verification stage to improve creative decision-making. 

These studies provide evidence of the existence of multilayered intuitive theories on emotion and creativity, involving creative or analytical processing in the task at hand (Cohen and Andrade 2004), stages of the creative act (Sas and Zhang 2010) and emotional personality traits such as neuroticism (Leung et al. 2014). Furthermore, the studies show that intuitive theories are being used spontaneously in emotion regulation that favors the creative process. Further research on these intuitive theories (the understanding of one’s emotions and their implications for creativity; an aspect of EI) as well as how they are used in the creative process (another facet of EI) is needed.

## 4. Collaborations

*Collaborations* refers to the involvement of others in the creative act, often to produce something that would not have been possible on one’s own (Lubart 2017). We will focus on the emotionally intelligent functioning and management of teams for enhancing creativity. 

Collaboration efforts benefit generally from members’ complementary skills, but being in complementary emotion states has also been shown to support creative output. Drawing on Dual-Tuning Theory (George and Zhou 2007), To and collaborators (2021) showed that the different cognitive processing styles favored by negatively and positively valenced emotion states benefit team creativity when occurring in different members over the same time period. Following a sample of 59 groups working on a creative project spanning 13 weeks, the authors measured *affect heterogeneity* (i.e., the extent to which team members reported being in positive as well as negative emotion states over the past week), team information exchange, as well as their *transactive memory system* (TMS), which includes role and expertise differentiation among team members as well as “a shared understanding of how to coordinate the flow of information to and from the right members” (To et al. 2021, p. 1232). Evidence was provided that affect heterogeneity leads to more creative team output via its effect on information processing. A positive mood state favors “broad and flexible thinking”, whereas a negative mood will favor “critical and persistent thinking”, both of which can be useful in creative teamwork when experienced by different members in a complementary way (p. 1231). However, the teams require adequate information exchange, which is supported by TMS, in order to take advantage of affect heterogeneity creatively. 

Given the close links between emotion and creativity, it is important to monitor and manage emotions in groups performing creative tasks (George 2000; Zhou and George 2003). Huy (2002) studied an organization undergoing “radical change” (during which a large number of new ideas were being implemented) and found that middle managers used emotion management in two ways that were successful for facilitating the process: maintaining motivation in themselves and others and attending to employees’ emotions. This allowed that “emotional intelligence could be created at the group level without requiring a majority of influential individuals to be emotionally intelligent” (p. 61). 

EI in leaders has predicted the creativity of group members in several studies (Rego et al. 2007; Castro et al. 2012; Ivcevic et al. 2021). Leaders’ self-control against criticism, empathy (Rego et al. 2007), self-encouragement and understanding of their own emotions (Castro et al. 2012) were identified as the most important dimensions of EI for creativity. When leaders are emotionally intelligent, Ivcevic et al. (2021) found that followers experience more positive affect and better opportunities to grow professionally, which in turn leads to increased creative output. EI in team members also increases creativity via the promotion of team trust and a collaborative culture (Barczak et al. 2010). It thus seems like EI “paves the way” for effective creative collaboration. When group members experience predominantly positive emotions (e.g., Ivcevic et al. 2021; Grawitch et al. 2003; Amabile et al. 2005) while also being in heterogeneous, complementary mood states (To et al. 2021) and when members feel that they can trust each other and their leaders as well as having opportunities for growth and effective collaboration (Ivcevic et al. 2021; Barczak et al. 2010), teams will be more likely to perform creatively. 

## 5. Contexts

The environmental conditions surrounding creativity can be both physical and social in nature (Lubart 2017; also see Harrington 2011). We will be examining the task as a context in interaction with emotion-related factors, including EI. We will also look at the role of EI during social evaluation.

A study from Abele in 1992 showed experimentally that the motivational influence of negative mood states on creativity is different according to how *interesting* the task is, i.e., that the task “elicits mainly positive or at least evaluatively neutral ideas” (Abele 1992, p. 206). An interesting creativity task will thereby have the potential to serve as *mood repair* when a person is in a negative mood state, which increases the motivation to exert an effort. Indeed, the participants in an induced negative mood outperformed the control group on creative fluency during the interesting unusual uses task, but had significantly lower results in the less interesting tasks (which were another unusual uses task as well as a fictional situation task). The valence of the generated ideas was altogether positive in the negative mood condition only when the task was interesting, which supports the mood repair hypothesis. Zenasni and Lubart (2011) extended this line of research by showing that the perceived pleasantness of creative idea-generation tasks increases ideational fluency. 

Another important aspect of creative tasks interacting with emotion is task framing. According to Friedman et al. (2007), a positive mood state will induce a motivation to seek stimulation. A task framed as congruent with this motivation (i.e., framed as being fun) will be performed more creatively as a result. When in a negative mood state, the induced motivation will be to solve problems, and a task with motivation-congruent potential (i.e., framed as being serious) will be performed with better results. Support for these hypotheses was found in three studies where identical creative idea-generation tasks were framed as being either serious or fun. Collectively, these findings can be used in emotionally intelligent ways to boost creative output, for example by consciously choosing the task to work on as a function of one’s mood state and/or the task’s intrinsic pleasantness. The ability to frame tasks requiring creativity in a mood-motivational congruent way could also depend on one’s level of EI. These are questions that call for further exploration. 

The social context surrounding the creative act can be stress-inducing when it involves social evaluation, which has an impact on creativity (Byron et al. 2010). The effects that negative and positive feedback have on creativity depend on people’s level of EI (Agnoli et al. 2019). Agnoli et al. (2019) used eye-tracking technology to measure affective arousal as well as the extent to which irrelevant stimuli were processed in a divergent thinking task. During the task, participants received either repeated positive or negative feedback, both of which led to increased affective arousal. It was found that affective arousal diminished fluency scores only in low-trait EI participants. In the negative feedback condition, participants high on trait EI showed an increase in the originality of responses when affective arousal was higher. The authors concluded that, with enough emotional resources, the stress of being evaluated can be managed in a way that creative performance is kept consistent. Moreover, the affective arousal that comes with negative feedback can be used to generate more original ideas when EI is higher. 

Furthermore, the same study found that fixating on irrelevant stimuli during the task led to a decrease in both fluency and originality in low-trait EI participants. However, it did not affect fluency in the high- trait EI group, even leading to an increase in originality. This could indicate that “irrelevant information becomes distracting for people overwhelmed by the stressful nature of the evaluations (i.e., low-trait EI individuals)”, whereas in high-trait EI individuals, irrelevant stimuli can instead be “used to obtain a larger pool of associations during ideational activities” (Agnoli et al. 2019, p. 247). Hence, evidence suggests that EI plays an important role when facing stressful aspects of the social context during a creative activity, both for managing affect and for broadening the attention span to creatively profit from elements of the physical environment.

## 6. Creations

*Creations* concerns the products resulting from the creative process (Lubart 2017). These can be tangible or not and can range from an idea that has not yet reached its final form to a fully finished creative product. In general, creative productions are original and useful and may lead to positive or negative consequences, being associated with positive or negative emotions. In this regard, Sternberg and Lubart (2022) have recently discussed positive, transformational creativity as contrasted with malevolent creativity, i.e., creations with harmful or immoral results (Cropley et al. 2008; Cropley et al. 2010). However, we consider this issue to be broader than the scope of emotion covered here, as consequences of creativity are not necessarily purely emotional in nature (for example, inflicting material or physical harm is not the same as inflicting emotional harm, though all three types would fall under malevolent creativity). Regarding the role of EI in malevolent vs. benevolent creativity, we refer the interested reader to Harris et al. (2013). 

In this section, we will review creative emotion regulation strategies as well as the effect of negative emotional states on judgments of creative products. Extensive work has been conducted specifically on cognitive reappraisal as a form of an emotion regulation strategy. In this context, cognitive reappraisal is defined as “changing the way that one thinks about a critical situation in order to alter its emotional impact” (Weber et al. 2014, p. 345). Weber et al. (2014) created the Reappraisal Inventiveness Test (RIT) to measure a person’s creativity or “inventiveness” (fluency and flexibility) in generating reappraisals. Four situations designed to evoke anger are presented sequentially. For example, in one situation you are asked to imagine yourself when a friend neglected to water your plants while you were away traveling, telling you upon returning to your dead plants that the distance to your apartment was just too long. Each participant is then allotted 3 minutes to generate as many categorically different reappraisals as possible for each situation. There is some evidence of reappraisal inventiveness being beneficial for well-being, for example predicting a lower number of depressive experiences in everyday life in men but not women in a student sample (N = 126) (Perchtold et al. 2019). 

Creativity in cognitive reappraisal and its potential benefits was also studied by Wu et al. (2017, 2019). The participants of an initial study (N = 31) were tasked with generating cognitive reappraisals for 25 negatively charged stimuli (Wu et al. 2017). Measures of self-assessed creativity and self-assessed effectiveness in regulating emotions of the generated reappraisals were found to be positively correlated. In subsequent studies, pretested cognitive reappraisals that were more or less creative were presented to the participants with regard to the negatively charged stimuli (Wu et al. 2017, 2019). Again, creative cognitive reappraisals were rated as significantly more effective than ordinary reappraisals (Wu et al. 2017). In addition, negatively charged stimuli that had previously been presented with creative cognitive reappraisals (as opposed to ordinary reappraisals or objective descriptions) were later experienced as significantly less unpleasant when presented without description (Wu et al. 2019). This finding applied not only during the same session but also three days later, indicating a longer-term effect (Wu et al. 2019). In sum, a number of studies seem to carefully point toward creativity in the domain of emotion regulation being potentially beneficial for psychological well-being.

Turning the perspective from the creation itself to its judgment, it is also important to consider emotional states when judging a creative product. After inducing either a positive, negative or neutral mood of equal valence, Mastria et al. (2019) administered the Alternative Use evaluation Task (AUeT) in which the participants (N = 48) rated a list of ideas on creativity. The ideas were pretested as being either noncreative, moderately or highly creative. Generally, participants in a positive mood rated the ideas as being more creative. For moderately creative ideas, the evaluations of participants in a negative mood state were significantly lower than the other groups. The findings partially support the authors’ hypothesis that the hedonic tone of a judge’s emotional state influences evaluations in a way that a negative state leads to risk aversion and a tendency to reject creativity, whereas a positive mood state makes the judge more inclined to “detect the quality of others’ ideas” (p. 3). In another study, Lee et al. (2017) found that inducing fear in participants made them judge two exogenous ideas as being significantly less creative than the ratings of happy as well as angry participants. The authors concluded that fear leads to uncertainty, which increases risk aversion, impacting evaluations of new ideas. More work is thus needed to elucidate the nature of emotional states’ effect on judges of creative ideas. Expanding this line of research, one could also examine the extent of emotional awareness that judges of creative products have, as well as whether or not they use this information to guide their actions (e.g., “I am in a bad mood today, so I better be careful as to how I judge”). These aspects of EI could play a role in a large variety of situations, from art contests to the rating of creative productions in research.

## 7. Consumption

Creative productions exist within a social context that may condition the willingness to endorse or use them. *Consumption* is the study of this issue, namely the adoption of creative ideas and productions (Lubart 2017). We provide some illustrations of how emotions can influence willingness to adopt new products, and we propose that this knowledge can be used intelligently to favor the adoption of new and innovative products. 

Aroean and Michaelidou (2014) report a study of 295 respondents who completed a questionnaire that included assessments of their *need for emotion* (their susceptibility to affective stimuli and situations), *consumer innovativeness* (their inclination to adopting innovative products) and hedonic enjoyment concerning a product of their own choosing. In situations eliciting enjoyment, innovative consumers were shown to be more “susceptible to promotional messages that contain emotional cues compared to cognitive messages”, i.e., having a greater need for emotion with regard to the adoption of new products (Aroean and Michaelidou 2014, p. 247). Horn and Salvendy (2009) found the participants’ ratings of positive affect in creative products as the only significant predictor of their willingness to purchase (i.e., adopt) the product. In studying willingness to adopt new products such as black toothpaste and uniquely flavored potato chips, Zhou et al. (2021) showed that when in a state of nostalgia, people experience a greater amount of social support, making them more inclined to adopt products that are new and unfamiliar to them. 

Using emotions intelligently to favor adoption could thereby be implemented, for example, by inducing a state of nostalgia in potential consumers (e.g., Zhou et al. 2021) or by using marketing to highlight the affective properties of innovative products (e.g., Aroean and Michaelidou 2014; Horn and Salvendy 2009). To our knowledge, the role of emotional intelligence for promoting the adoption of creative products and innovation has not previously been studied. This could be a potentially fruitful line of research for marketing or consumer behavior.

## 8. Curricula

*Curricula* concerns the cultivation of creativity, for example, through specific techniques or educational programs (Lubart 2017). The question asked in this section is the following: Does training people’s EI or related abilities help develop their creativity? 

One group of researchers developed training programs for children (Hoffmann et al. 2021), adolescents (Maliakkal et al. 2016), families (Maliakkal et al. 2017) and professional adults (Ebert et al. 2015) designed to teach EI and creativity skills using the arts as a medium. These workshops consisted of 6-8 sessions with different themes in which skills such as perspective taking, identifying and reflecting upon emotions and engaging in unconventional thinking were taught via specific art-related activities. Apart from the children’s workshop, in which participants showed a significant but short-lived increase in ideational fluency after the intervention in both a problem finding and an alternate uses task (Hoffmann et al. 2021), the studies were evaluated using qualitative methods. The sample of adolescents (N = 37) reported, among other things, being better able to use metaphors and symbols to describe feelings after the intervention (Maliakkal et al. 2016). 

Another recent study administered a variant of a Design and Technology (D&T) course including empathy instructions and focusing on real-world problems (Demetriou and Nicholl 2022). The participants were 13–14-year-old students from two different schools (N = 64), where one group received the normal D&T lessons and the other received the modified teaching program during one semester. The effect that empathy had on creativity is described as an increased ability to take the perspective of others when suggesting original solutions to problems. Pre- and postintervention assessments using the Torrance Test of Creative Thinking (TTCT) showed an increase in both cognitive and empathic aspects of creativity, including a greater total creativity score in the intervention-school at the end of the semester but not before (Demetriou and Nicholl 2022). The total emotion score of the TTCT (a combination of emotional expressiveness and expressiveness of titles) was also significantly greater in the intervention-school after receiving the training, but not before the intervention. 

Finally, Drama Pedagogy Training (DPT) and creative drama instruction have also been used to teach emotion and creativity skills (Célume et al. 2019; Yeh and Li 2008). Yeh and Li (2008) measured the extent to which 116 children from different preschools had been exposed to creative drama instructions, including activities such as “making use of fantasy, doing role plays and pretend plays, imitating movements, acting in dramatic plays, and telling stories” (p. 135). They found that children’s creativity, as measured by performance on a specially adapted *Preschooler’s Creativity Test* (PCT), was positively correlated with the amount of creative drama instruction that they received. DPT shares similarities with creative drama and is, in its adoption by Célume et al. (2019), focused on “playing creative-collaborative dramatic games” and “the creative and socio-emotional competencies that might be learnt collectively” in this way (Célume et al. 2019, p. 2). After a total of six sessions, the children who received the DPT intervention scored higher on EPoC (Lubart et al. 2011) divergent but not convergent thinking and furthermore reported being in a more positive and stable mood (Célume et al. 2019). 

To answer the above-stated question, there is indeed some initial empirical support that the training of emotion skills and resources goes hand in hand with enhanced creativity. These findings strengthen the evidence of the connection between EI and creativity. 

## 9. Discussion and Conclusions

Using the seven Cs of creativity as a structuring framework (Lubart 2017), we have provided illustrations of how work on EI concerns each facet of creativity research. Some of the Cs (notably creators and collaborations) have been more explored than others, and some Cs are starting to receive attention in emerging research (e.g., curricula and creations). Yet other Cs connections to EI and related aspects have been illuminated by a few promising but isolated studies, insofar not receiving concentrated, systematic attention (such as creation, contexts and consumption). We have also provided suggestions of possible future directions in the context of each individual C. 

Finally, the use of the seven Cs framework allows us to design research that looks at a combination of two or more Cs. For example, studies could look at creators x context. In this type of study, individual differences in emotional intelligence together with variations in contextual emotions may interact, such that individuals with a high EI are more creative in general, but particularly so in a negative emotional context compared to individuals with low EI (in a similar vein as Agnoli et al. 2019). Also using a creators x context design, Mikulincer and Sheffi (2000) assessed the adult participants’ attachment style (creators) and had them perform several tasks, including the RAT, while being in a positive vs. negative mood (context). Similarly to Leung et al. (2014), researchers could also look at emotion-related individual differences and how these affect the way that emotions are monitored and used in the creative process in a creators × creating design. Moreover, research has examined the creativity as well as the content of participants’ creations in terms of its darkness–brightness (Cropley et al. 2010) in various emotional contexts, such as positive vs. negative mood induction (Yefet and Glicksohn 2021) or in situations/tasks with aggressivity-inducing instructions and cues vs. ones designed as benevolent (Harris and Reiter-Palmon 2015). Hao et al. (2017) used positive vs. negative emotion induction (context) while having the participants perform divergent thinking tasks in an open vs. closed body posture (creating) to find whether there was an interactive effect when emotion and posture were congruent (i.e., open–positive, closed–negative). As these examples illustrate, several C × C study designs can be found in the literature. Looking forward, using the seven Cs of creativity as a framework could allow us to go further. Table 1 presents hypothetical research designs in each C in potential combinations with all the other Cs. It is designed to illustrate the usefulness of the seven C taxonomy for generating new hypotheses and testing them in interacting C × C designs.

For future endeavors, there could be studies of creators × curricula showing aptitude-treatment interactions (ATI), with people who have low EI benefiting for creativity from a specific emotion-related training, whereas people with initially higher EI levels benefit for creativity from a different type of emotion-related training (see Table 1). A creating × collaborations research design could also be envisioned, in which a team’s creative process is traced as well as the affective dimension, for example, assessing the perception and regulation of emotions in oneself and others during creative collaboration. Study designs could also incorporate more than two Cs. For example, participants with a high- vs. low-trait alexithymia (creators) could be tasked to generate ideas for a happy vs. sad situation (contexts) while working either alone or collectively in groups (collaborations). We believe that these sorts of multiple C designs allow for nuanced exploration of the complex relationship between emotion and creativity and could be especially useful for understanding the way that emotions are perceived, understood, managed and used intelligently for creativity. 

This review suggests that there is a great potential to do more work on EI, EC and related abilities in conjunction with creativity. We hope that this seven Cs taxonomy can help connect and stimulate future research endeavors.

## Figures and Tables

**Table 1:** C × C study designs. Creators: Level of trait EI (2 groups—high vs. low); Creating: Process tracing of emotions in a creative act; Collaborations: Working alone or together creatively; Contexts: Emotionally laden or emotionally neutral creativity task; Creations: Generating strategies to regulate emotion creatively, such as cognitive reappraisal; Consumption: Assessing willingness to adopt original products and level of emotional content that the products produce; Curricula: Training of EI and creative skills.

	Creators	Creating	Collaborations	Contexts	Creations	Consumption
Creating	Examining the emotional process flow chart in high- vs. low-trait EI individuals during a creativity task					
Collaborations	Do high- vs. low-trait EI individuals perform better creatively in groups vs. alone?	While performing a creativity task, is there a difference in the succession of emotions in groups vs. alone?				
Contexts	Do high- vs. low-trait EI individuals perform better creatively in emotionally laden or emotionally neutral tasks?	Examining the creative process during an emotionally laden vs. emotionally neutral creativity task	Are emotionally laden vs. emotionally neutral tasks resolved more creatively when working in a dyad or alone?			
Creations	Are high- vs. low-trait EI individuals more effective in generating original and efficient cognitive reappraisals?	Are there different emotional process profiles in individuals generating more or less creative cognitive reappraisals?	Are there benefits to receiving support by others when regulating emotions creatively? - A cognitive reappraisal task performed either alone or in a dyad	Do people generate more creative emotion regulation strategies faced with more or less negative situations?		
Consumption	Does perceived emotional content in creative products predict willingness to adopt the product, and is there an interaction with level of trait EI?	Tracing judges’ level of emotions while they choose to adopt or dismiss presented creative products	Do dyads vs. separate individuals experience more or less affect when faced with a creative product and does that affect their willingness to adopt it?	Does the emotional context of a product’s use mediate the willingness to adopt a creative product?	Are people who regularly use creative emotion regulation strategies more inclined to be influenced by affective appeals when adopting creative products?	
Curricula	Do individuals high vs. low on trait EI benefit differently from an EI/creativity training program?	Evaluating if EI/creativity training affects individuals’ emotional process during a creative act (comparing before and after the training)	Is EI/creativity training more successful when given to individuals separately or in dyads?	Evaluating if EI/creativity training affects individuals’ performance on emotionally laden vs. emotionally neutral creativity tasks	Can EI/creativity training improve individuals’ proficiency in generating creative cognitive reappraisals?	Does EI/creativity training impact individuals’ habits of consuming creative products? Is this related to the level of emotions perceived when faced with the products?
